# Quantification of Sunscreen Ethylhexyl Triazone in Topical Skin-Care Products by Normal-Phase TLC/Densitometry

**DOI:** 10.1100/2012/807516

**Published:** 2012-05-02

**Authors:** Anna W. Sobanska, Jaroslaw Pyzowski

**Affiliations:** Department of Analytical Chemistry, Medical University of Lodz, Ulica Muszynskiego 1, 90-151 Lodz, Poland

## Abstract

Ethylhexyl triazone (ET) was separated from other sunscreens such as avobenzone, octocrylene, octyl methoxycinnamate, and diethylamino hydroxybenzoyl hexyl benzoate and from parabens by normal-phase HPTLC on silica gel 60 as stationary phase. Two mobile phases were particularly effective: (A) cyclohexane-diethyl ether 1 : 1 (v/v) and (B) cyclohexane-diethyl ether-acetone 15 : 1 : 2 (v/v/v) since apart from ET analysis they facilitated separation and quantification of other sunscreens present in the formulations. Densitometric scanning was performed at 300 nm. Calibration curves for ET were nonlinear (second-degree polynomials), with *R* > 0.998. For both mobile phases limits of detection (LOD) were 0.03 and limits of quantification (LOQ) 0.1 **μ**g spot^−1^. Both methods were validated.

## 1. Introduction

**Figure 1:**
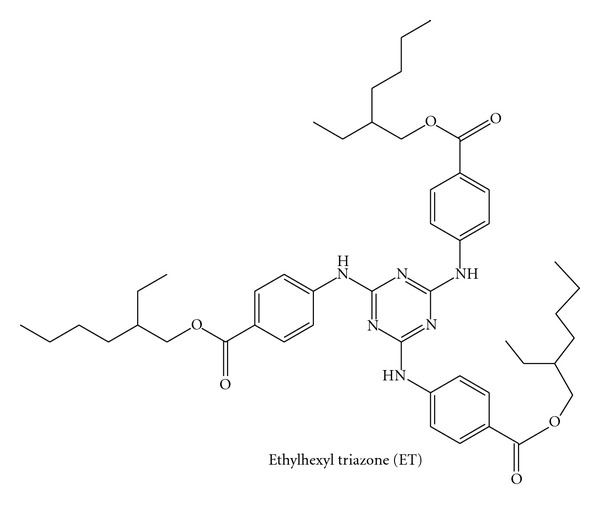
Structural formula of ET.

Ethylhexyl triazone (ET, Figure 1) is an oil-soluble UVB filter (*λ*_max⁡_ in ethanol 314 nm) manufactured by BASF under the trade mark Uvinul T150 and used in cosmetic formulation at concentrations up to 5%. Due to its insolubility in water and affinity to the skin keratin, it is particularly suitable for water-resistant products. Its excellent photostability and high absorption coefficient make it a valuable ingredient when a high SPF (sun protection factor) value is required [1]. 

Ethylhexyl triazone was quantified in cosmetic product mainly by RP-HPLC [2–9], less frequently UPLC [9], or UV spectrophotometry [10]. HPLC is also the technique of choice in ET skin permeation studies *in vitro* [11–13] as well as in the analysis of environmental samples containing ET [14, 15]. Liquid chromatography was usually performed on RP-18 [2–4, 7, 9, 11–14] or RP-8 [5, 15] stationary phases and coupled with UV [2–9, 11–14] or MS [15] detectors.

The objective of this study was to develop a simple and cost-effective method of analysis of ethylhexyl triazone in complex sunscreen preparations by normal-phase thin-layer chromatography followed by densitometry.

## 2. Experimental

### 2.1. Chemicals, Material and Solutions

Uvinul T150 (ethylhexyl triazone) was kindly donated by BASF. Cyclohexane, diethyl ether, acetone, ethyl acetate, toluene, isopropanol, and methanol were from Polskie Odczynniki Chemiczne (POCh), Poland. SPF 20 water-resistant sun-care lotion containing ethylhexyl triazone, avobenzone, and octocrylene (Sample A) was manufactured by DAX Cosmetics, Poland. SPF 30 sun-care moisturizing cream containing ethylhexyl triazone, diethylamino hydroxybenzoyl hexyl benzoate, and octyl methoxycinnamate (Sample B) was from Soraya, Poland. Both cosmetic products analyzed throughout this study were preserved with parabens.

Uvinul T150, 500 mg, was weighed accurately into 100-mL volumetric flask, dissolved in adequate amount of acetone and diluted to volume to give a stock solution of the concentration 5 mg mL^−1^. The stock solution of ET was diluted with acetone to prepare standard solutions (0.1, 0.2, 0.4, 0.6, 0.8, 1.0, 1.2, 1.4, 1.6, 1.8, and 2.0 *μ*g *μ*L^−1^).

### 2.2. Sample Preparation

Sun-care products (1000 mg) were weighed accurately into 100 mL volumetric flasks. Approximately 70 mL methanol was added to each sample, and the flasks were vigorously shaken by use of a Premed (Poland) type 327 Universal Shaker for 60 min. Methanol was then added to volume, and the flasks were wrapped with aluminum foil and left to stand for 60 min.

### 2.3. Thin-Layer Chromatography

Thin-layer chromatography was performed on 10 × 10 cm HP quality silica gel 60 plates (layer thickness 0.2 mm) from Merck or on 10 × 20 cm standard quality silica gel 60 plates (layer thickness 0.25 mm), also from Merck. Plates were spotted with the Desaga AS 30 sampler equipped with a 10 *μ*L syringe (1 *μ*L spot^−1^), 15 mm from the bottom edge and at 8 mm intervals, starting 10 mm from the plate edge and developed with either cyclohexane-diethyl ether 1 : 1 (v/v), Method A, or cyclohexane-diethyl ether-acetone 15 : 1 : 2 (v/v/v), Method B. Plates were developed in a vertical chromatographic chamber lined with filter paper and previously saturated with the appropriate mobile phase vapor for 20 min. Development distance was 75 mm from the plate bottom edge. After development, plates were dried at room temperature (20°C), scanned, and analyzed in reflectance mode with the Desaga CD 60 densitometer at 300 nm.

### 2.4. Analysis of Ethylhexyl Triazone in Sunscreen Creams or Lotions

The sunscreen products solutions in methanol, prepared as described above, were spotted on silica gel 60 HP TLC plates (2 *μ*L). The plates were then chromatographed as described above for ET standards (Section 2.3).

## 3. Results and Discussion

### 3.1. Method Development

The sun-care preparations analyzed in this study contained, apart from ET, other UV filters, that is, avobenzone (AVO) and octocrylene (OCR) (Sample A) or octyl methoxycinnamate (OMC) and diethylamino hydroxybenzoyl hexyl benzoate (DHHB) (Sample B), and preservatives absorbing within the UV range (parabens). In the course of our earlier research [16–18], three stationary phases (silicagel 60, RP-2 and RP-18) and several mobile phases were investigated. ET is a relatively lipophilic compound with strong affinity to RP-18 stationary phase [16–18]. On the other hand, its separation from AVO, OCR, OMC, and DHHB on RP-2 stationary phase is poor [16]. For these reasons, it was decided that silica gel 60 is the stationary phase of choice. Mobile phases capable of effective ET separation from other UV filters listed above included cyclohexane-diethyl ether 1 : 1 (v/v), cyclohexane-diethyl ether-isopropanol 15 : 1 : 1 (v/v/v), toluene-ethyl acetate 15 : 1, and cyclohexane-diethyl ether-acetone 15 : 1 : 2 (v/v/v) [16–18]. An additional requirement was, however, that ET and sunscreens such as AVO and OCR (Sample A) or OMC and DHHB (Sample B) should be separated by one chromatographic procedure prior to their simultaneous densitometric quantification. This was achieved in the case of Sample B with mobile phase cyclohexane-diethyl ether-acetone 15 : 1 : 2 (v/v/v) on silica gel 60 [18] (Method B). In the case of Sample A, separation of ET from AVO and OCR was also successful (silica gel 60, cyclohexane-diethyl ether 1 : 1 (v/v), Method A), but simultaneous quantification of AVO and OCR required a different approach because these sunscreens coelute under most conditions [16, 17]. Both mobile phases gave ET spots of sufficient quality for densitometric analysis, although in the case of mobile phase B the spots were of slightly better quality.

Analytical wavelength suitable for ET analysis (300 nm) was selected on the basis of multiwavelength scans obtained for this sunscreen.

**Figure 2:**
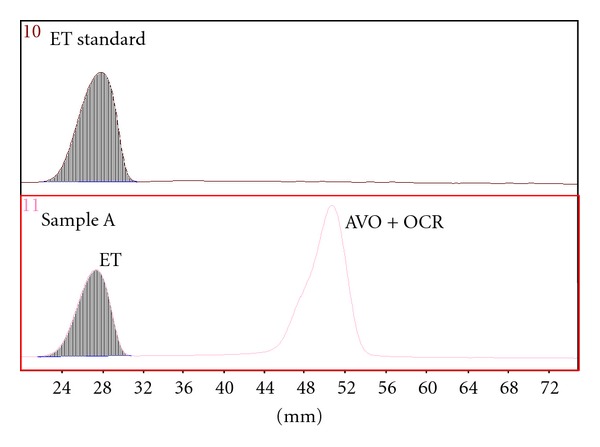
Densitograms of Sample A and ET standard (300 nm), Method A.

**Figure 3:**
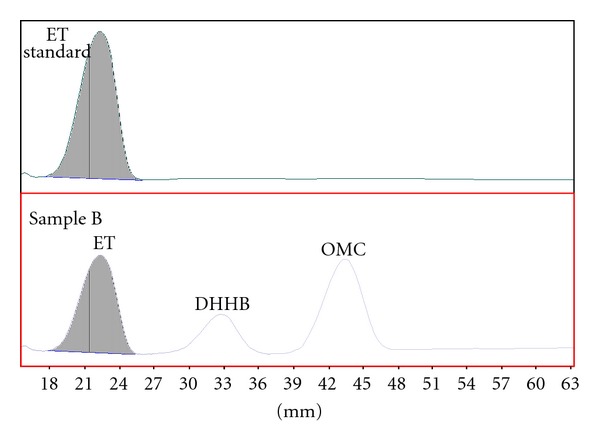
Densitograms of Sample B and ET standard (300 nm), Method B.

Typical densitograms of ET separated by Methods A and B are presented in Figures 2 and 3.

### 3.2. Method Validation

#### 3.2.1. Specificity

Sun-care preparations analyzed throughout this study contained, apart from ET, other ingredients absorbing within the UV range such as OCR, AVO, DHHB, OMC, and parabens. *R*_*f*_ values for compounds of interest are as follows:

- mobile phase A: *R*_*f*_(ET) 0.20, *R*_*f*_(AVO) 0.55, *R*_*f*_(OCR) 0.60, *R*_*f*_(ethylparaben) 0.30, *R*_*f*_(OMC) 0.53, *R*_*f*_(DHHB) 0.37 [16, 17];
- mobile phase B: *R*_*f*_(ET) 0.10, *R*_*f*_(AVO) 0.42, *R*_*f*_(OCR) 0.44, *R*_*f*_(ethylparaben) 0.15, *R*_*f*_(OMC) 0.47, *R*_*f*_(DHHB) 0.30 [16, 18].

**Figure 4:**
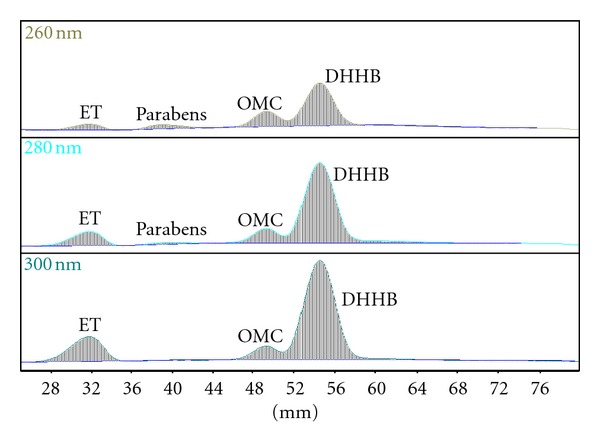
Multiwavelength scans of Sample B (260–300 nm), cyclohexane-diethyl ether-isopropanol 15 : 1 : 1 (v/v/v).

Chromatographic conditions A and B are suitable for separation of ET from UV filters listed above and, according to our earlier studies [16], from the majority of other sunscreens used in contemporary sun-care preparations. The efficiency of Method B is slightly lower since the separation of ET from parabens is incomplete (*R*_*f*_ values for ET and ethylparaben are 0.10 and 0.15, resp.); this is, however, not a problem, since the analytical wavelength for ET is 300 nm (Section 3.1.), and, as it can be seen in Figure 4, parabens do not absorb at 300 nm.

**Figure 5:**
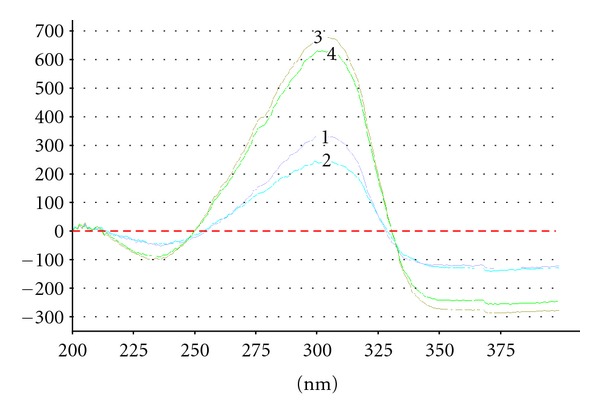
UV spectra of ET isolated from Sample A (1, 2, 3) and of ET standard (4).

Purity of ET peaks obtained during the analysis of Sample A was confirmed by UV/VIS spectra of sunscreens acquired directly from chromatographic plates in reflectance mode. Spectra collected at three different points of particular peaks obtained for the sample solution were compared with spectra acquired for the standard (Figure 5).

#### 3.2.2. Calibration

**Figure 6:**
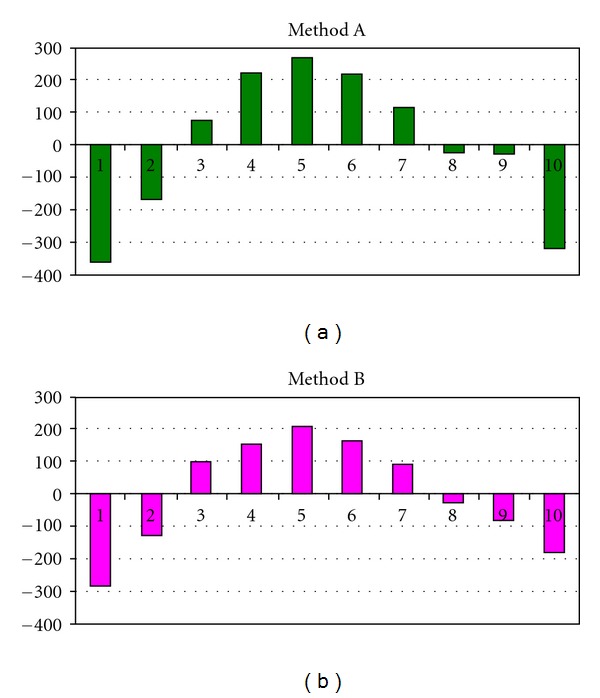
Residues test for calibration plots A and B, linear regression. Method A: *y* = 2341.5*x* + 874.84, *R* = 0.9905. Method B: *y* = 1409.5*x* + 763.97, *R* = 0.9851.

**Figure 7:**
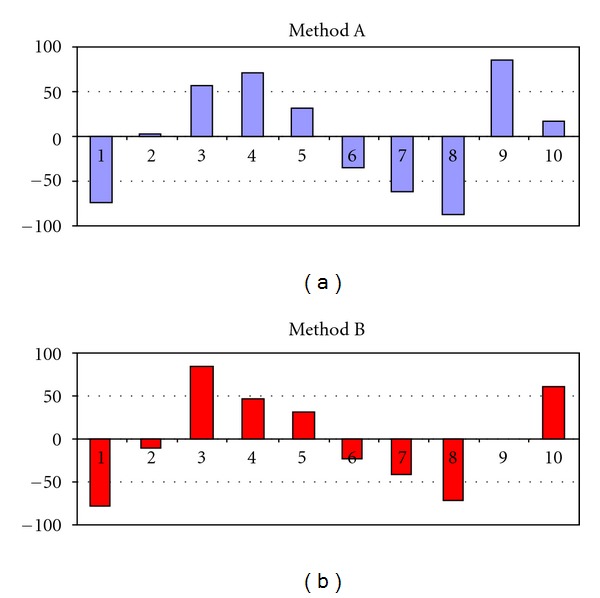
Residues test for calibration plots A and B, second-degree polynomials (equation according to Table 1).

Calibration plots for Methods A and B were obtained by plotting peak areas against amount of ET in the range 0.1–2.0 *μ*g spot^−1^. In both cases linear regression coefficients were relatively high (*R* = 0.9905 and 0.9851, resp.) but since this should not be used as the sole proof of linearity, nonnumerical analysis of residues according to [19] was performed. Residues (differences between experimental values and those calculated on the basis of appropriate equations) for linear calibration plots proposed for methods A and B showed strong tendencies which suggested that linear fit is inappropriate (Figure 6). Two possibilities were considered at this stage: selecting a narrower, pseudolinear range or using a different type of equation. Calibration plots were finally generated in the form of second-degree polynomials (Table 1), and their quality was assessed again by means of *R* values and non-numerical analysis of residues (Figure 7). Residues plots for quadratic calibrations A and B (Figure 7) showed the lack of tendency that combined with very high *R* values confirmed the correctness of curves fitting. It should be mentioned in this point that densitometric detection in Methods A and B was performed in reflectance mode. Lambert-Beer's law cannot be applied to diffuse reflectance so calibration in TLC/densitometry is seldom perfectly linear [19]; if this is the case, quadratic equations are often used [19].

#### 3.2.3. Precision

Repeatability of the method was tested according to [19–21] by replicating the entire method on the same day, using the same cosmetic preparations, batches of solvents, and chromatographic plates, by the same analyst (Day 1, Analysis I and II). Intermediate precision was verified according to [19–21] by repeating the procedure on the same cosmetic preparations but on a different day, by a different analyst, using other batches of solvents and chromatographic plates (Day 2). The results of these experiments (Table 2) prove that the methods' precision is sufficient for routine product analysis.

#### 3.2.4. Limits of Detection and Quantification

The limits of detection and quantification for ET determined experimentally on the basis of signal-to-noise ratio according to [22] are given in Table 1.

#### 3.2.5. Robustness

After due consideration of factors that can influence the analysis results, it was concluded that the critical points are the quality of chromatographic plates (HPTLC versus TLC) and the method of spotting. The same cosmetic preparations were analyzed on HPTLC silica gel 60 chromatographic plates with automatic spotting and on standard TLC silica gel 60 plates with manual spotting with a microsyringe. The results of these analyses (Table 2) are similar, but coefficients of variations are slightly higher for manual spotting.

Additionally, the influence of small, deliberate changes in the mobile phase compositions on the results of ET quantification was tested:

Method A: cyclohexane-diethyl ether 0.9 : 1.1 (v/v)
Method  B: cyclohexane-diethyl ether-acetone 15 : 0.9 : 2.1 (v/v/v).

The results of these changes are summarized in Table 2.

#### 3.2.6. Accuracy

Blank cosmetic creams were spiked with ET, AVO, and OCR (A) or ET, OMC and DHHB (B) at three concentrations 1, 3, and 5% (w/w) of each sunscreen corresponding to 0.2, 0.6, and 1.0 *μ*g spot^−1^ (2 *μ*L spot^−1^ of the cream solution prepared according to Section 2.2.). The analytical procedures A and B described in Section 2 were performed on the samples, and the recoveries are presented in Table 3.

#### 3.2.7. Storage and Stability of Standard Solutions

Standard solutions of ET as well as solutions of other sunscreens and preservatives used in this investigation were refrigerated between the experiments and not exposed to light except for time needed for plate spotting. The stability of all solutions was in these conditions excellent as tested by UV/VIS spectroscopy over the period of 2 weeks.

## 4. Conclusions

Ethylhexyl triazone may be quickly and effectively separated from other oil-soluble UV filters and preservatives by normal-phase HPTLC on silica gel 60. Separation can be achieved by a variety of mobile phases, of which two, cyclohexane-diethyl ether 1 : 1 (v/v) or cyclohexane-diethyl ether-acetone 15 : 1 : 2 (v/v/v), were found superior. The methods of ethylhexyl triazone separation and quantification presented in this paper are based on one of the cheapest stationary phases (silica gel 60, compared e.g., to RP-18 or RP-8 layers) and do not require toxic solvents. The analyses may be performed with analytical-grade solvents (HPLC purity solvents are not required), and, although HPTLC plates and automatic spotting are preferred, relatively good results may be achieved on standard-quality TLC plates spotted with a microsyringe. Fast, reliable and cost-effective densitometric quantification of ET proposed in this paper may, therefore, be recommended for routine analysis of cosmetic products.

## Figures and Tables

**Table 1:** Calibration plots for ET: Methods A and B, automatic spotting, 300 nm.

	Method A	Method B
Equation	*y* = −640.2*x* ^2^ + 3656.8*x* + 469.0	*y* = −474.72*x* ^2^ + 2384.8*x* + 463.0
*R*	0.9993	0.9983
LOD [*μ*g spot^−1^]	0.03	0.03
LOQ [*μ*g spot^−1^]	0.1	0.1

**Table 2:** Results of repeatability, intermediate precision, and robustness tests.

	HP TLC plates, automatic spotting (*n* = 3), 2 *μ*L spot^−1^				TLC plates, manual spotting (*n* = 3), 1 *μ*L spot^−1^	Modified mobile phases (*n* = 3)
		Day 1, Analyst 1		Day 2, Analyst 2		
		Analysis A	Analysis B			
Sample A Method A	*μ*g spot^−1^	0.46	0.48	0.48	0.22	0.47
	% in formulation	2.30	2.40	2.40	2.20	2.35
	CV%	1.8	1.7	2.2	4.1	1.5

Sample B Method B	*μ*g spot^−1^	0.19	0.20	0.21	0.10	0.20
	% in formulation	0.95	1.00	1.05	1.00	1.00
	CV%	2.7	2.1	2.5	5.1	2.3

**Table 3:** Recovery tests (*n* = 3), 2 *μ*L spot^−1^.

% in formulation (w/w)		Method A	Method B
1.0	Found (%)	0.95	1.00
	% recovery	95.0	100.0
	CV%	5.0	4.3

3.0	Found (%)	3.15	2.90
	% recovery	105.0	96.7
	CV%	4.9	3.9

5.0	Found (%)	4.80	5.10
	% recovery	96.0	102.0
	CV%	4.5	4.2
